# Immunohistochemical Characterization of the Chemosensory Pulmonary Neuroepithelial Bodies in the Naked Mole-Rat Reveals a Unique Adaptive Phenotype

**DOI:** 10.1371/journal.pone.0112623

**Published:** 2014-11-19

**Authors:** Jie Pan, Thomas J. Park, Ernest Cutz, Herman Yeger

**Affiliations:** 1 Department of Paediatric Laboratory Medicine, The Hospital for Sick Children, Toronto, Ontario, Canada; 2 Developmental and Stem Cell Biology, Research Institute, The Hospital for Sick Children, Toronto, Ontario, Canada; 3 Physiology and Experimental Medicine, Research Institute, The Hospital for Sick Children, Toronto, Ontario, Canada; 4 Department of Biological Sciences, University of Illinois at Chicago, Chicago, Illinois, United States of America; 5 Department of Laboratory Medicine and Pathobiology, University of Toronto, Toronto, Ontario, Canada; Faculty of Animal Sciences and Food Engineering, University of São Paulo, Pirassununga, SP, Brazil, Brazil

## Abstract

The pulmonary neuroepithelial bodies (NEBs) constitute polymodal airway chemosensors for monitoring and signaling ambient gas concentrations (pO_2_, pCO_2_/H^+^) via complex innervation to the brain stem controlling breathing. NEBs produce the bioactive amine, serotonin (5-HT), and a variety of peptides with multiple effects on lung physiology and other organ systems. NEBs in mammals appear prominent and numerous during fetal and neonatal periods, and decline in the post-natal period suggesting an important role during perinatal adaptation. The naked mole-rat (NMR), *Heterocephalus glaber*, has adapted to the extreme environmental conditions of living in subterranean burrows in large colonies (up to 300 colony mates). The crowded, unventilated burrows are environments of severe hypoxia and hypercapnia. However, NMRs adjust readily to above ground conditions. The chemosensory NEBs of this species were characterized and compared to those of the conventional Wistar rat (WR) to identify similarities and differences that could explain the NMR’s adaptability to environments. A multilabel immunohistochemical analysis combined with confocal microscopy revealed that the expression patterns of amine, peptide, neuroendocrine, innervation markers and chemosensor component proteins in NEBs of NMR were similar to that of WR. However, we found the following differences: 1) NEBs in both neonatal and adult NMR lungs were significantly larger and more numerous as compared to WR; 2) NEBs in NMR had a more variable compact cell organization and exhibited significant differences in the expression of adhesion proteins; 3) NMR NEBs showed a significantly greater ratio of 5-HT positive cells with an abundance of 5-HT; 4) NEBs in NMR expressed the proliferating cell nuclear antigen (PCNA) and the neurogenic gene (MASH1) indicating active proliferation and a state of persistent differentiation. Taken together our findings suggest that NEBs in lungs of NMR are in a hyperactive, functional and developmental state, reminiscent of a persistent fetal state that extends postnatally.

## Introduction

The subterranean dwelling naked mole-rat (*Heterocephalus glaber*; NMR) has been gaining increasing attention by biologists since it has been recognized to possess several remarkable and often unusual properties compared to other rodents and humans [1]–[3]. NMRs are extremely long lived, with a lifespan exceeding 28 years (versus 4 years for mice), and show extraordinary resistance to cancer [4], [5], inflammation [6], acid [7], [8], ammonia [9], pain related behaviors [6], and aging [2], [6]. Recent studies have shown that the remarkable resistance of NMR to cancer is due to the secretion by fibroblasts of an extremely high molecular mass hyaluronan (HA) with decreased activity of HA degrading molecules and a unique HA synthase [4]. Knock-down of the HA synthesis enzyme, HAS2, or overexpression of the enzyme degrading HYAL2 made the fibroblasts susceptible to transformation and tumorigenicity [4].

The eusocial subterranean lifestyle with large colony numbers, high humidity, hypoxia and hypercapnia suggests other unique physiological adaptations, and indeed neural tissues of NMRs show high tolerance to hypoxia [10]–[12]. Resistance to oxygen nutrient deprivation for 24 hours was demonstrated in hippocampal slices [10], and adult NMR retain a protective, neonatal-like NMDA receptor subunit profile [13]. Remarkably too, NMRs do not exhibit evidence of extracellular plaques, nor an age-related increase in amyloid beta peptides (Ab) despite the relatively high content and having a one amino acid difference from human Ab [14].

The effects of a harsh external microenvironment on other cells and organs such as lung, is as yet not known. The observation that the lungs in NMRs are tractable to extremely different environmental conditions (hypoxic/hypercapnic burrows and conventional laboratory animal housing) [15] raised the question about lung chemosensory functions in this species. We have been studying the chemosensory functions in lung as mediated by the pulmonary neuroendocrine system, PNEC, and related multicellular neuroepithelial bodies (NEBs) and have characterized these specialized cells and their physiological responses to hypoxia and hypercapnia [16]. Thus we asked if the NEBs in NMRs were similar to other species or exhibited unique features given this species’ extreme hypoxia/hypercapnic microenvironment, since NEB hyperplasia has been described following experimental chronic hypoxia and in high altitude dwellers [16]. This is important as the O_2_/CO_2_ sensing function of NEBs is still being debated by some investigators favoring NEB function as mechano-or pain sensors [17]. In fact pain sensing is highly reduced in NMRs [18]. Since NMRs live under hypoxia/hypercapnia, but are adaptable to being raised under normoxic conditions, this stimulated our investigation of PNEC/NEBs in NMRs. The animals studied here were raised under normoxic conditions in an animal facility. We examined NMRs at different ages with respect to NEB structure and expression of neuroendocrine markers. Here we report that NEBs in NMRs are similar in many ways to conventional rodents, express marker epitopes readily detected in human NEBs, and also exhibit some interesting differences in marker expression that could explain their ability for adaptation to low ambient O_2_ and high CO_2_ concentrations. Interestingly, the overall composition of the NEB chemosensor in NMRs resembles the human O_2_ chemosensor complex.

Finally, NEB cell immunophenotyping suggests that in the NMR NEBs, these cells exist in a fetal-like transitional developmental state which may underlie their adaptive plasticity. These observations highlight the value of NMRs as a potential model for the study of human relevant chemosensory functions.

## Methods

### Animals and Tissues

Lung tissues from naked mole-rats (n = 8) (NMR; *Heterocephalus glaber*) at postnatal day 5–3 months were embedded in polyethylene glycol (OCT medium) (Lab-Tek Products; Naperville, IL). Wistar rats (n = 4), used as a conventional animal control, at postnatal day 3–8 months, were obtained from Charles River (St. Constant, Quebec) and housed in the Hospital for Sick Children lab animal facilities. All animal procedures were in accordance with Canadian Council of Animal Care guidelines and were approved by the Animal Care and Use Committee of the Hospital for Sick Children. Adult rats were sacrificed by lethal injection of euthanyl and neonates by cervical dislocation. Dissected lungs were washed three times in CO_2_-independent medium and embedded in OCT and then snap frozen on dry ice. All tissue blocks were sealed and stored at −80°C until use.

### Immunofluorescence Labeling

Cryosections of the medial segment of the middle lobe from NMR and Wistar rat (WR) lungs were cut at 60–80 µm under low working temperature (−12 to −15°C). The sections were immediately transferred to a dish with zinc formalin fixative (Newcome Supply; Middleton, WI) at RT. After three changes of fresh fixative (10 min each at RT), the sections were washed in PBS and stored at 4°C for 2 weeks maximally. For immunoflourescence labeling studies a variety of primary and secondary antibodies were used with the type, source and dilutions listed in Table 1. Dual immunofluorescence labeling (antibodies to *gp91^phox^* plus SV2 antibodies/*p22^phox^* plus SV2) was performed on sections permeabilized with 1% Triton X-100 in PBS for 10 min and then blocked in 20% normal donkey serum in 4% BSA plus avidin/biotin blocking solution for 60 min at RT. Following several brief washes slices were incubated with selected pairs of primary antibodies (e.g. mouse anti-SV2 mAb mixed with rabbit anti-pannexin1 pAb or rabbit anti-synaptophysin mAb mixed with mouse anti-MASH1 mAb) observing different host species (Table 1), at dilutions shown, and at 4°C overnight on an orbital shaker. To enhance relative weaker signals of SV2 and SYP antibodies, the biotin-secondary antibody conjugate (against mouse or rabbit) plus streptavidin-Texas Red X conjugate were applied during the procedure for dual immunolabeling. Indirect immunoperoxidase method for various neuroendocrine markers was used for the demonstration of PNEC/NEBs in sections of paraffin embedded NMR and WR lungs. Sections (5 µm) were deparaffinized and rehydrated through descending alcohol series and in PBS. For antigen retrieval, sections were treated with 10 mM sodium citrate buffer (pH 6.0; Sigma) and endogenous peroxidase quenched with 0.03% hydrogen peroxide (Fischer) in PBS for 10 min. After application of primary antibodies the immunostaining procedure was performed following the manufacture’s instruction for application of SuperPicture 3^rd^ Gen IHC Detection Kit (Invitrogen).

**Table 1:** Primary and Secondary Antibody Sources and Working Dilutions.

Primary antibodies	Dilutions	Sources
Rb anti-Synaptophysin mAb (SYP)	1∶20	NeoMarkers
Ms anti-Synaptic vescle protein 2 mAb (SV2)	1∶100	Hybridoma Bank
Gt anti-Serotonin pAb (5-HT)	1∶500	DiaSerin
Ms anti-Smooth muscle actin-FITC mAb	1∶250	Sigma
Ms anti-N-CAM mAb	1∶100	Abcam
Ms anti-E-cadherin mAb (E-CA)	1∶200	Zymed
Rb anti-ZO1 pAb	1∶200	Zymed
Rb anti-Pannexin 1-N pAm	1∶300	Invitrogen
Gt anti-clera cell 10 Kd protein (CC10)	1∶500	Santa Cruz Biotech
Ms anti-Proliferating cell nuclear antigen mAb (PCNA)	1∶100	Novus
Ms anti-MASH1 mAb	1∶250	BD Pharmingen
Ms anti-CGRP mAb	1∶300	Invitrogen
Rb anti-gp91*^phox^*/NOX2 pAb	1∶1500	Abcam
Rb anti-p22*^phox^* pAb	1∶2000	Santa Cruz Biotech
Rb anti-Carbolic anhydrase II pAb	1∶2000	Abcam
Rb anti-P_2×2_ pAb	1∶1000	Abcam
Rb anti-Vesicular acetylcholine transporter (Vacht) pAb	1∶1000	Chemicon
Rb anti-Vesicular monoamine transporter 1 (VMaT1)pAb	1∶1500	Santa Cruz Biotech
Rb anti-nitric oxide synthase (cholinergic NOS) pAb	1∶1000	Abcam
Rb anti-K*v*3.3 (KCN3) pAb	1∶800	Alomone Lab
Rb anti-K*v*4.3 (KCN4) pAb	1∶500	Alomone Lab
Ms anti-HIF1 α mAb	1∶200	Abcam
Rb anti-HIF2α pAb	1∶500	Abcam
**Secondary antibodies**	**Dilutions**	**Sources**
Anti-mouse IgG H+L-biotin	1∶200	Jackson Laboratories
Anti-rabbit IgG H+L-biotin	1∶200	Jackson Laboratories
Anti-goat IgG H+L-biotin	1∶400	Jackson Laboratories
Anti-rabbit IgG H+L-FITC	1∶100	Jackson Laboratories
Anti-mouse IgG H+L-Texas Red	1∶100	Jackson Laboratories
Streptavidin-Texas Red X	1∶1000	Invitrogen
HRP-Polymer-anti IgG conjugate	ready to used	Invitrogen
RedDot-2 dye (697nm)	1∶200	Biotium

### Confocal Microscopy

Fluorescent immunolabeling images of PNEC/NEBs, airway nerves, and smooth muscle in the double-stained whole mount slices were obtained with a Leica confocal laser scanning microscope (model TCS-SPE) and LAS-AF software. The variable excitation wavelengths of the krypton/argon laser were 488 nm for FITC, 568 nm for Texas Red and 695 nm for RedDot 2 (nuclear staining).

### Morphometric Analysis

For quantification of NEBs in NMR lungs we used a method similar to that for mouse lung as previously reported [19]. We measured the integrated surface area of bronchioles of different sizes, expressed in square millimeters of the section (5 µm/100 µm thickness) using the NIH-Image J program standardized by an internal scale bar in each acquired image in each counted confocal image. The numbers and sizes of NEBs were assessed in three sections from the middle lobe and immunostained for SV2 or SYP. The total number of NEBs and PNECs in each section was divided by the integrated surface area and the relative number expressed as a mean ±SEM per mm^3^ of lung tissue based on calculated volume of three 10 µm frozen sections. To determine the ratio (%) of serotonin positive cells among cells staining for pan-neural markers SV2/SYP marking NEBs; 5-HT positive cells and SV2/SYP stained cells were manually counted in all 45–50 µm thick sections dual immunolabeled confocal images. The individual ratios of 5-HT positive cell numbers to total SV2/SYP positive cells from two size NEB groups (>40 µm and <40 µm) were calculated [19].

### Statistical Analysis

One-way analysis of variance (ANOVA) with repeated measures was used for statistical analysis of NMR lungs and rat lungs with respect to the different stages in development. One-way ANOVA tests with repeated measures were also used for comparison of NEB numbers and integrated density of immunostaining in NMR lung and rat lung. All data are expressed as means (+/−) standard error of the mean (SEM).

## Results

### Synopsis

Neuroendocrine markers were used to identify PNEC/NEBs in NMR airways, and the antibodies were used to delineate structural similarities and differences are listed in Table 1. Table 2 summarizes the immunostaining results and shows a comparison between NMR and postnatal WR (when NEB numbers are maximal) in terms of relative expression levels for all marker antibodies listed in Table 1 and with respect to staining of NEBs, nerves, epithelium and smooth muscle in the respective lungs. The information here pertains to the subsequent discussions and highlights both clear differential staining and differences in intensity of expression. What stands out is the broad level of positive antibody reactivity shown in NMR lung tissues versus the WR suggesting either definitive expressions and/or greater accessibility of epitopes.

**Table 2:** Summary of immunoreactivities of antibodies comparing postnatal to 3 month naked mole-rat lungs with postnatal rat lungs.

AntibodyAgainst:	Naked Mole Rat Lung				Rat Lung			
	NEB	Nerve	Ep*	SM**	NEB	Nerve	Ep*	SM**
SV2	+++	+++	–	–	+++	+++	-	-
SYP	+++	+++	–	–	+++	+++	–	–
CGRP	+++	–	–	–	+++	–	–	–
5-HT	++	–	–	–	+	–	–	–
NCAM	+	+++	–	–	–	–	–	–
E-CAM	+	–	+++	–	–	–	+	–
CNX43	+++	–	++	++	+	–	++	++
Pannexin 1	++	–	+++	–	–	–	–	–
CC10	–^S^	–	+++	–	ND	ND	ND	ND
ZO-1	+	–	+++	–	–	–	+++	–
gp91*^phox^*	++	–	+	–	++	–	++	–
p22*^phox^*	++	–	+	–	–	–	–	–
CA-II	++	–	+	–	–	–	–	–
VChT	+	+++	–	–	–	+	–	–
VMat	++	–	–	–	+	–	–	–
Kv3.3	+++	++	–	++	–	–	–	–
Kv3.4	+++	++	–	+++	–	+	–	++
iNOS	+	+++	–	–	–	±	–	–
*P_2×2_*	+	+++	–	–	+	++	–	–
PCNA	+#	–	–	–	ND	ND	ND	ND
Mash1	+#	–	–	–	ND	ND	ND	ND
HIF-1α	++	–	–	–	ND	ND	ND	ND
HIF-2 α	++	–	–	–	ND	ND	ND	ND

*Ep = airway epithelium; **SM = airway smooth muscle; # = nuclear expression; ^S^ = small number of CC10-positive cells associated with NEBs; ND = not determined.

### NEB features and neuroendocrine markers

**Figure 1:**
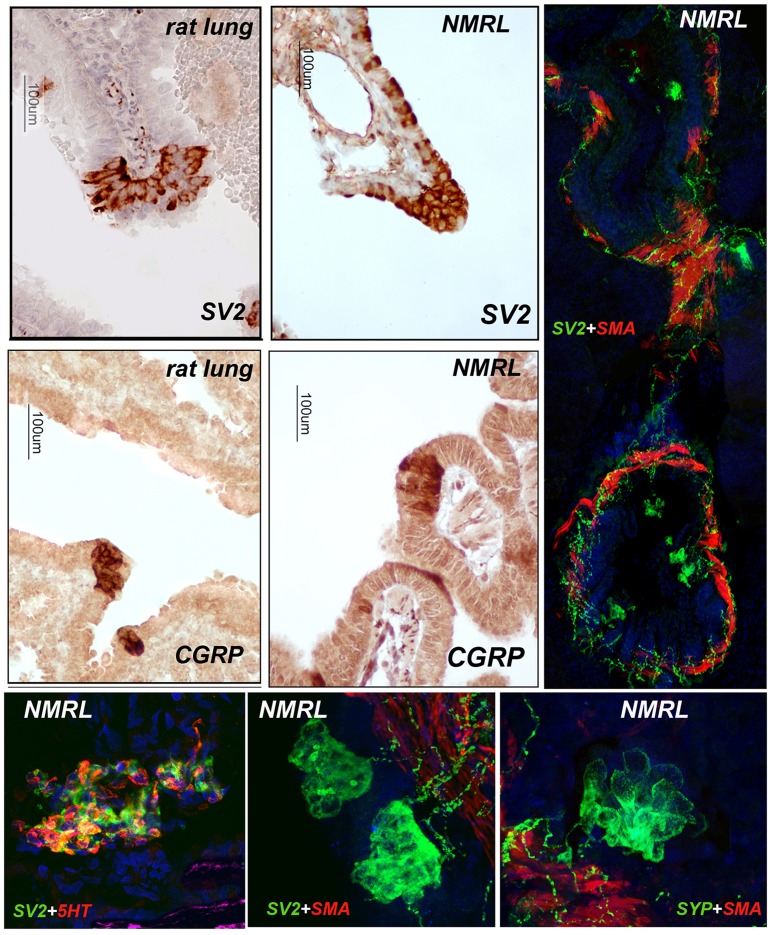
Neuronal related neuroendocrine markers in NMR NEBs. A] Immunohistochemical staining of WR (left side) NMR lung (right side) showing strong staining of NEBs in different airways for SV2 and CGRP. B] IF labeling for SV2 reveals an extensive network of nerve fibers and occasional submucosal ganglia innervating the muscle surrounding airways (SMA positive, red) and NEBs (SV2 positive) located within the epithelium. C] Co-labeling for 5-HT and SV2 reveals the large size of NMR NEBs and heterogeneity in 5-HT expression within the NEBs. D] A higher magnification of NEBs stained positive for SV2 reveals the number of nerve fibers running through submucosal smooth muscle (SMA, red) and also innervating the muscle. E] Note that the NMR NEBs strongly express synaptophysin (SYP) correlating with the neural relevant expression of SV2.

**Figure 2:**
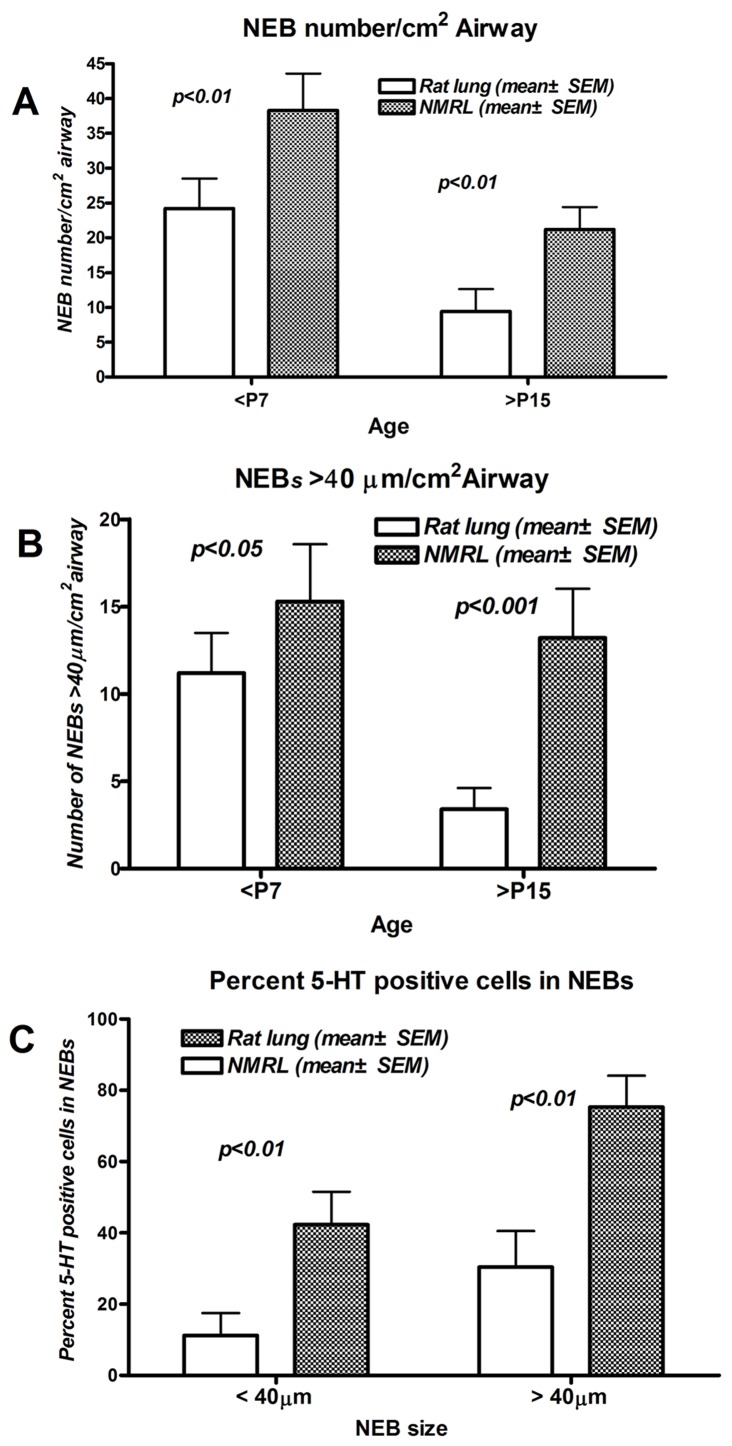
Morphometric analysis of NEBs in NMRL vs rat lung. Comparison between lungs older than postnatal day 15(>P15) and less than postnatal day 7(<P7). A&B) In every case NEB numbers, size, and 5-HT positive cells within NEBs are significantly higher in the NMRL. Note also that NEB numbers and size decrease somewhat after the neonatal period. C) Ratio of 5-HT positive cells in NEBs>and<than 40 um. Ratios determined versus postnatal day 7 lungs. Larger airway NEBs show a higher ratio of 5-HT positive cells both in NMR and rats. This confirms previous observations in airways in different species. Notably, the NMR NEBs exhibit a significantly higher content of 5-HT positive cells. Results expressed as mean+/− SEM.

Immunohistochemical staining shows that NMR NEBs in the postnatal to 3 month age range can be easily identified by strong expression of pan-neural markers SV2 and CGRP outlining the individual cells in the NEBs (Figure 1). Whereas by immunohistochemistry SV2 also stained single cells, CGRP expression was more restricted to NEBs. Immunofluorescence labeling with antibodies to SV2 and SMA (smooth muscle actin) definitively outlined the NEBs and extensive arrays of nerve fibres innervating both the SMA labeled smooth muscle and the NEBs. NEBs and nerves also stained prominently for expression of synaptophysin. Morphometric analysis revealed that NMR NEBs, enumerated as NEBs/cm^2^ airway, were significantly more numerous by ∼2 fold over that in WR (Figure 2). NEB size was similarly increased in NMR compared to WR and the differential staining increased during the postnatal period (Figure 2). Of real interest is the staining pattern obtained with antibody to 5-HT (serotonin) showing strong expression of 5-HT in many but not all cells within the NMR NEB cluster. The ratios of 5-HT positive cells to 5-HT negative cells calculated for NMR and WR show a significantly greater proportion of 5-HT positive cells for the NMR (Figure 2). The staining patterns and quantification give first indication that NMR NEBs are prominent features of the NMR lung which anatomically is also less complex (fewer lobes) than in other rodents (not shown). Furthermore, we noted variability in the compactness of many NMR NEBs compared to other species where NEBs appear more compact [17]. This last observation led us to ask if NMR NEB cells expressed adhesion proteins found in previous studies.

### Cell adhesion markers

**Figure 3:**
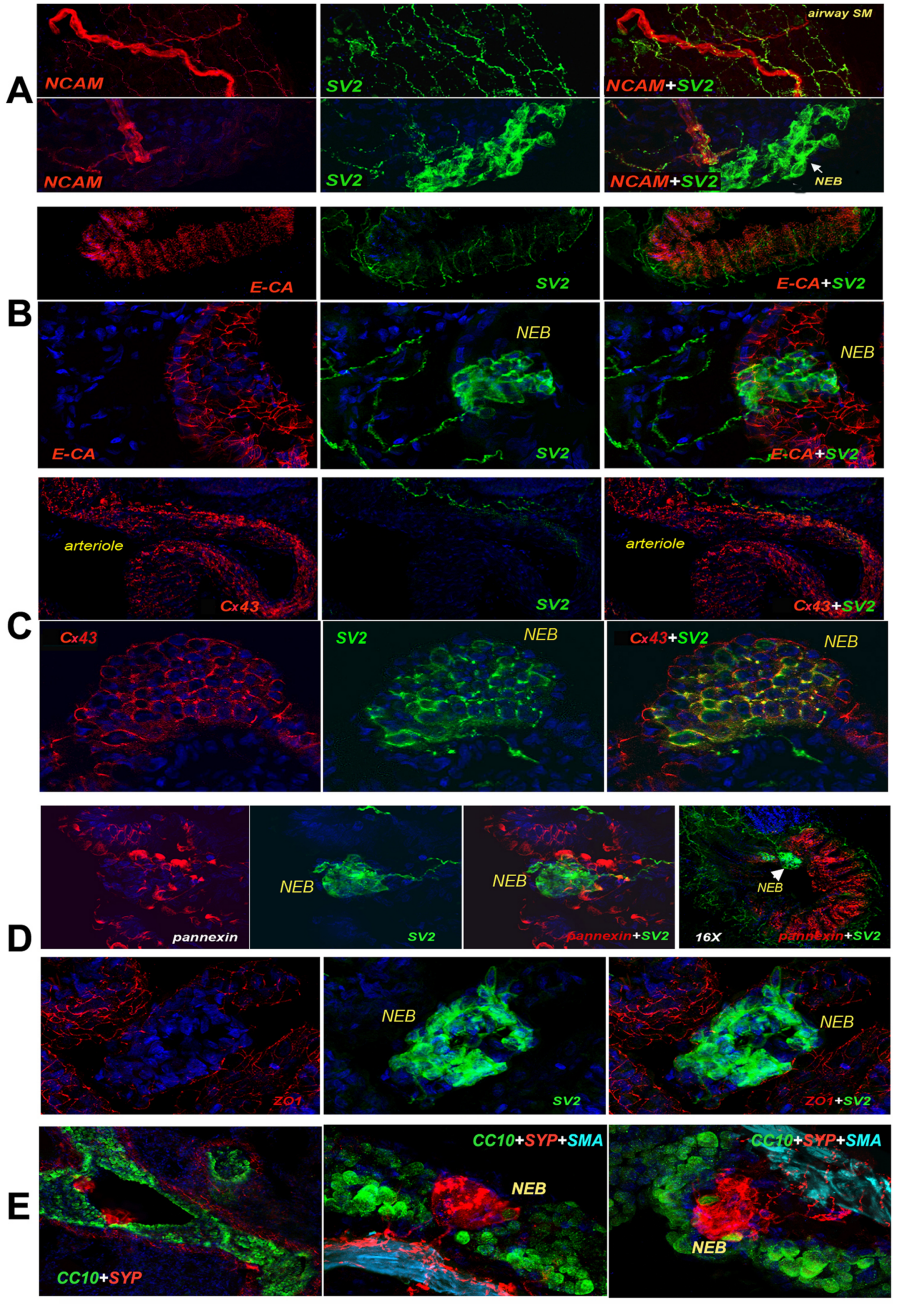
Adhesion molecules/communication junctions of NMR NEBs. A large panel of markers were used to phenotype the lung parenchyma with respect to cell adhesion markers (NCAM, E-Cadherin, ZO-1, Cx43) relative to SV2 marking the NEBs and nerves. A] Showing that large nerve fibers are labeled for NCAM, while small nerve fibers label for SV2. Note that SV2 labels a large NEB with relatively loose arrangement of PNEC within the NEB. NEB staining for NCAM is relatively weak or negative amongst cells. B] E-cadherin identifies the airway epithelium which is penetrated by many fine nerve fibers stained for SV2. Note that E-cadherin is weakly expressed within the NEBs (plane of section may only indicate epithelial cell protrusions) while SV2 only labels nerves and NEBs. C] In reference tissue types, Cx43 strongly labels arterioles but not SV2. In contrast, NEB cells strongly express both Cx43 and SV2 in a coordinated pattern. D] Pannexin1 was expressed strongly by the epithelial cells surrounding SV2 labeled NEBs. Few NEB cells expressed pannexin1. Interestingly, NMR NEB cells are not connected via tight junctions as compared to the ZO-1 positive surrounding epithelial cells. E] Staining for CC10 (Clara cell marker) in NMRL reveals an abundance of CC10 positive cells residing within the NMR airway epithelium. Note a small number of CC10 positive cells are located within the NEBs or are tightly associated with NMR NEB cells identified by staining positively for synaptophysin (SYP). SMA staining identifies the underlying smooth muscle.

To determine if species differences in cell adhesion were associated with the less compact phenotype for NMR NEBs, we stained for the neural cell adhesion molecule, NCAM. We found that SV2 positive NMR NEBs barely expressed NCAM in comparison to surrounding nerves (Figure 3A). This is different than in other species where NEBs stain strongly or equivalent to nerves for NCAM [17]. Staining for the epithelial adhesion protein, E-cadherin, showed that, as expected, the surrounding epithelium stained positively for E-cadherin. In stark contrast, the NMR NEB cells barely showed immunoreactivity for E-cadherin (Figure 3B). The comparative strong immunoreactivity for SV2 also depicted connecting nerves. Any trace of E-cadherin signal likely appeared due to projections from interspersed epithelial cells.

At another level of cell-cell communication we examined expression of the gap junction protein, Connexin 43 (Cx43) (Figure 3C). Anti-Cx43 strongly and uniformly labeled the NMR NEBs suggesting a robust degree of gap junction cell-cell communication. In addition, arteriole smooth muscle cells utilize, as expected, Cx43 for communication and coordinating functions. However, in contrast again to the surrounding epithelium, when examining for tight junctions using the ZO-1 marker, we observed that NMR NEB cells were not tightly connected through ZO-1 as compared to the surrounding epithelium (Figure 3D). By the same token examining for expression of newly identified hemichannel protein, pannexin (related to connexins), that modulates ATP release [20], we found that NMR NEB cells, in contrast to the surrounding epithelium, barely expressed this molecule (Figure 3D). In comparison, as reference, we stained for the Clara cell protein CC10 and found a highly robust expression in the surrounding epithelium (Figure 3E) resembling that seen in other rodents. As CC10 is involved in airway regeneration and innate immunity [21], the NMR respiratory epithelium might exist in a strong anti-inflammatory and regenerative state. Interestingly, the presence of a small number of CC10 positive cells, either residing within NEBs or closely adjacent to NEB cells, suggests that these could represent the stem cell progenitors as previously reviewed [22]. Thus the investigation of cell adhesion and cell-cell communication molecules suggests that the apparent less compact organization in some NEBs is supported by the apparent paucity of cell adhesion molecules that would enable a tighter and more compact organization. Nevertheless, the NMR NEB cells do appear to utilize gap junctions for communication which would facilitate a more uniform signaling response. Therefore we next examined if NMR NEB cells were competent in chemosensing in their expression of the key proteins of the NADPH oxidase complex and CO_2_ sensing carbonic anhydrase [17].

### O_2_/CO_2_ sensor complexes

**Figure 4:**
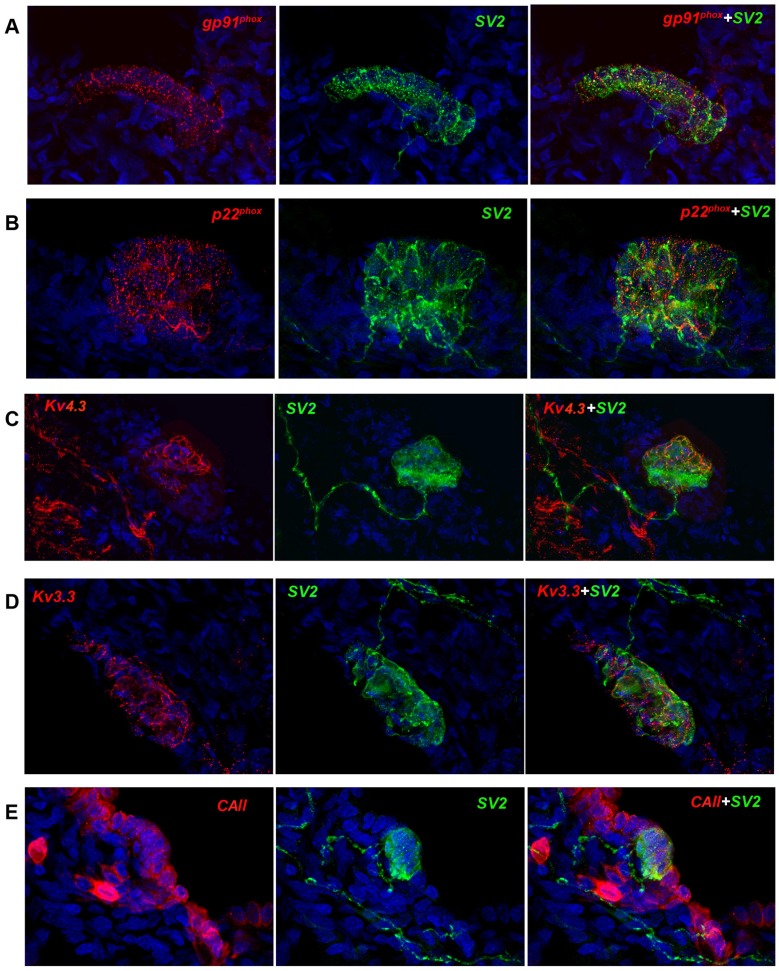
O_2_ and CO_2_ sensing related proteins and O_2_ sensitive K^+^ channels. Only the NEB cells express the NADPH oxidase proteins A] gp91^phox^ and B] p22^phox^ at the cell membrane at a high level suggesting a robust chemosensory function. SV2 marked the NEB cells and correlated closely with NADPH oxidase protein staining. C&D] The chemosensory function of NEBs includes specific K^+^ channels working cooperatively. Note that both Kv4.3 and Kv3.3 are richly expressed by NMR NEB cells located at the plasma membrane and directed towards the airways. E] The major cytoplasmic carbonic anhydrase, CAII, is shown to be expressed by epithelial cells and the NEB cells as well as potential submucosal nerve bodies, but not submucosal muscle.

In previous studies we extensively described the expression and temporal-spatial organization of the O_2_ sensing NADPH oxidase chemosensory complex at the apices of NEB cells [17]. We therefore asked if NMR NEB cells, given that in nature they are exposed to a hypoxia/hypercapnia environment, also expressed this O_2_ sensing complex and the degree of expression. We found a very uniform and robust expression of gp91^phox^ and p22^phox^ by NMR NEB cells (Figure 4A,B) suggesting a highly active O_2_ sensing state. Since the NADPH oxidase complex works coordinately with specific potassium channels, eg Kv 4.3 and Kv3.3, to signal the hypoxia state, we examined their expression. We found again that expression levels of Kv4.3 and Kv3.3 matched that of gp91^phox^ and p22^phox^ (Figure 4C,D) thus completing the O_2_ sensor complex and supporting the notion of a highly sensitive and active O_2_ sensing mechanism in NMR NEB cells.

More recently we have reported that CO_2_ sensing in NEBs is likely carried out with the CO_2_ responsive enzymes, carbonic anhydrases [Livermore et al, submitted]. We stained for the major cytoplasmic carbonic anhydrase (CA), and found CAII expression in NMR NEB cells occurred to a lesser degree than that in the surrounding epithelium (Figure 4E). Whether NMR NEB cells utilize other CAs for CO_2_ sensing has yet to be determined. Thus it may be that the CA profile in CO_2_ responsive NEB cells could change with respect to ambient conditions.

### Markers of innervation

**Figure 5:**
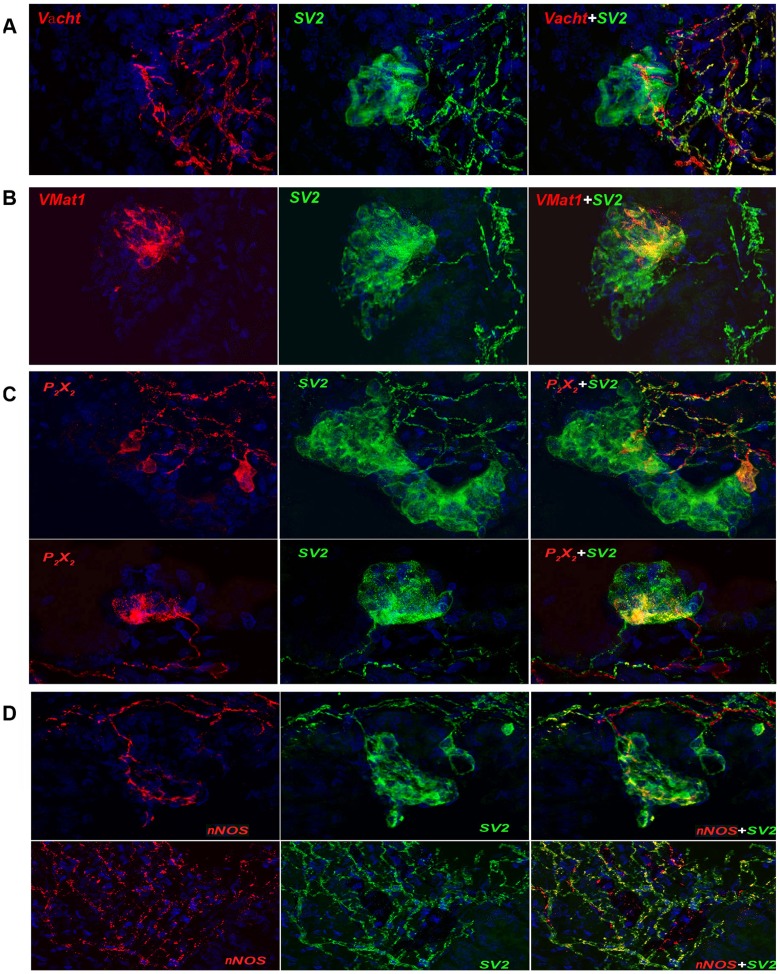
Expressions of cholinergic, purinergic and nNOS markers in NEBs of NMRL. The innervation of NMR NEBs was investigated for presence of cholinergic, purinergic and nitrergic inputs. A] Cholinergic innervation was extensive in the nerve network subtending the NEBs and a few fibers were found to make contact with NEBs at the base, or B] in selected areas (VMat1). C] Cholinergic fibers indicating extensive purinergic innervation subtended the NEBs and contacts appeared to be made through a few cells only. Note that individual purinergic P_2×2_ rich cells are seen within the NEBs. D] Nitrergic innervation was limited but nitrergic fibers penetrated deeper into the NEBs. In other areas nitrergic fibres were abundant as marked by nNOS.

We used SV2 staining to discriminate NEBs in previous figures and it is also apparent that SV2 delineated a rich innervation network in NMR NEBs. The current evidence in the field indicates that NEBs are innervated by both efferent and afferent nerves, with the afferent vagal nerve providing the feedback pathway to the brainstem [17]. Innervation appears to be mediated by adrenergic, purinergic and nitrergic fibres in varying proportions dependent upon the species [23], [24]. We therefore stained for Vacht, VMat1, P_2×2_ and nNOS as markers of these neural fibres (Figure 5A–D).

The immunostaining results (Figure 5 A,B) show that Vacht and VMat1 positive fibers enter at the NEB base making contact with some cells. The purinergic P_2×2_ fibers are different in that specific fibers connecting with neural P_2×2_ cells are part of the NMR NEBs suggesting specialized mediators. The limited number of P_2×2_ strongly positive cells suggest intraNEB specialization (Figure 5C). An abundance of nNOS positive nitrergic fibers were seen in the airways with a small number of contacts within the NMR NEB cells along one side (Figure 5D). This suggests specialization of neural signaling within the NEB cell complex and potentially differential sensory specializations of NMR NEB cells. Thus these results make it conceivable that a single NEB is comprised of different subpopulations of cells specialized in multiple sensory functions and potentially eliciting different mediators. These mediators may include the amines and different neuropeptides expressed by the neuroendocrine cells and other neurotransmitters such as ATP [17], [21]. Here we have already shown expression of 5-HT and CGRP.

**Figure 6:**
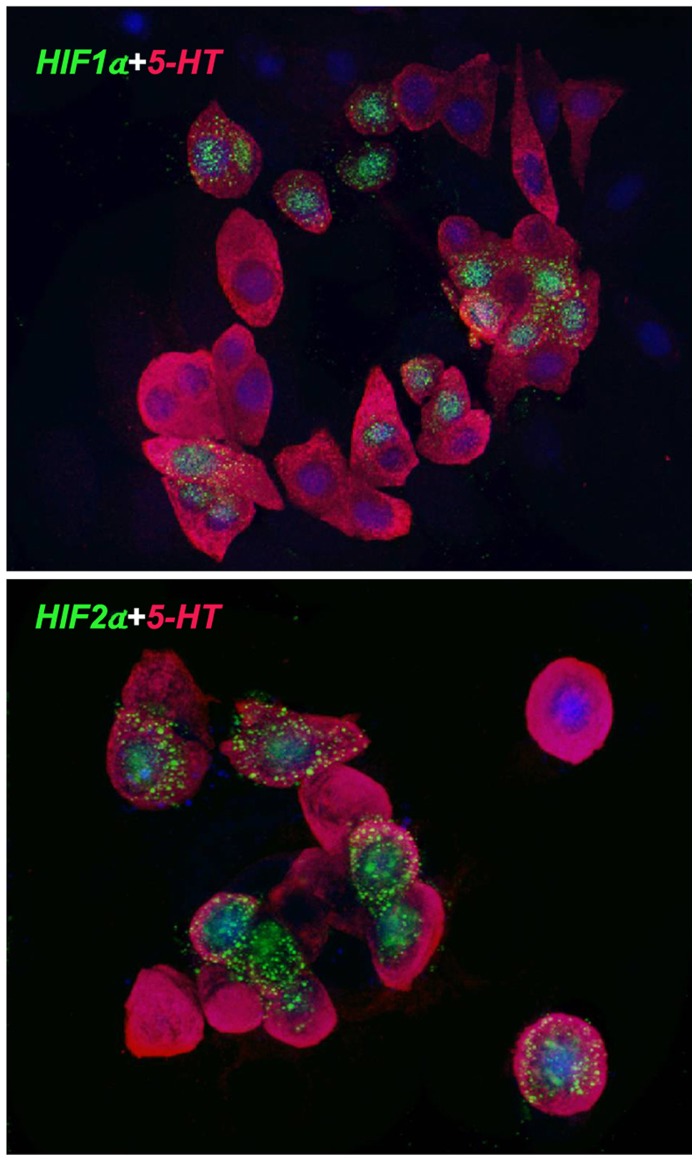
HIF1α and HIF2α in cultured NEBs of NMR. Primary cultures of NMR lungs were established short term and immunostained for 5-HT to identify NEB cells and coordinately for HIF1α and HIF2α. Note the strong nuclear staining for HIF1α (top) and mostly cytoplasmic staining for HIF2α (bottom). Note also that not all cells immunostain for these hypoxia sensitive markers suggesting different physiological states for cells within the NEBs and potentially different sensory capabilities.

This idea of specialization within the NMR NEB cells begged the question whether these cells were equally responsive to hypoxia. We therefore stained the NMR NEBs for expression of HIF1α and HIF2α, key hypoxia inducible proteins that mediate hypoxia driven transcriptional events in most cells. For these studies we examined both lung sections and primary cultures where moderate hypoxia often prevails under the relatively deep zone of medium. The *in vitro* culture of NMR tissues, other than fibroblasts, is currently under development so we will report on this aspect subsequently [manuscript in preparation]. Our findings here using short term primary cultures (Figure 6) reveal that little if any expression of HIFs was observed in native lung (not shown). However, in primary cultures a subpopulation of 5-HT positive NEB cells were strongly nuclear positive for HIF1α while other 5-HT positive cells were negative (Figure 6). In comparison, a subpopulation of NMR NEB cells were HIF2α expressing but mainly cytoplasmic. This differential staining suggests that *in vitro* HIF1α is activated transcriptionally but not overtly for HIF2α. These observations again suggest heterogeneity amongst the NMR NEB cells, and potentially a more complex neuroendocrine organ. To further examine this plasticity we examined the developmental state of the NMR NEBs.

### Proliferative potential and neurogenic gene expression

**Figure 7:**
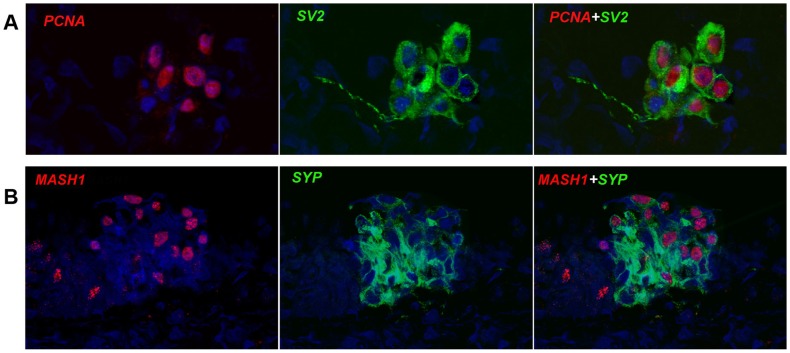
Proliferation and neurogenesis markers of NEBs in NMRL. NEB cells were examined for their intrinsic proliferative state and neurogenic status. In A] PCNA nuclear staining indicates that some NEB cells are cycle ready while in B] a proportion of NEB cells are positive for nuclear MASH1. This together suggests a more fetal like developmental state. Both robust SV2 and synaptophysin expressions indicate a functional neuroendocrine status of NMR NEB cells.

The results obtained thus far suggested that NEB cells in NMRs possess a fair degree of functional maturity. It has been well established that PNEC/NEB development is dependent on the obligatory expression of the neurogenic gene transcription factor, ASH-1 (hASH1 in humans, MASH1 in mice) and that expression of this transcription factor diminishes and disappears as NEBs mature [25], [26]. In the case of the NMR, its adaptability to hypoxic/hypercapnic environments and ease for switching to normoxic environments could imply a highly flexible phenotypic and developmentally plastic state. We stained for the proliferation marker PCNA (proliferating cell nuclear antigen) and the neurogenic marker MASH1 (Figure 7). Remarkably, we found that many NEB cells in adult NMR exist in a ready proliferative on state, since cells in G_o_ also mark with PCNA. Equally remarkable, prominent MASH1 nuclear staining was obtained in most of the NEB cells suggesting that in the adult NMR a significant proportion of cells within the NEBs also exist in a developmentally pliable state as well suggesting more of a fetal character.

It was not possible at this time to determine if the cells not staining for PCNA were also negative for MASH1 suggesting that these could be functionally terminal. Taken together with the other observations it would appear that the NMR NEB cells, being in a postnatal advanced age, occupy a developmental state seen in the neonatal and prenatal period in other species.

## Discussion

This first exploration of the pulmonary neuroendocrine cell system in the NMR has highlighted several unique differences between the NMR and other rodents and interestingly has indicated that epitopes recognized by the different antibodies more closely align with those in humans. Here we show that antibodies that do not readily work in other rodents easily worked with NMR lung tissues. We found that the NMR NEB cells were more variably compact in their organization compared to WR and other species, and probably communicate via the gap junction protein Cx43. On the other hand, NCAM, a homophilic binding protein, and ZO-1, the tight junction protein, appeared to be expressed minimally, and perhaps only on interspersed non-NEB cells. Barring possible alterations in NMR of unique detectable epitopes, this finding supports the idea that the neuroendocrine cells within NMR NEBs maintain a weaker cell-cell association which might permit greater ability to reorganize functionally. This then could be surmised as potential developmental plasticity leading to adaptability.

The robust expression of SV2, CGRP, synaptophysin and 5-HT essentially maximized on the expression levels for these markers as seen in NEBs in general, but were equally abundant in the NMR NEB cells. By comparison, whereas mouse only expresses 5-HT at a low to moderate level, human and rabbits have relatively high levels of 5-HT [17].

The finding that NMR NEB cells are competent in expression of the expected O_2_ chemosensing proteins, gp91^phox^ and p22^phox^, at appreciably high levels, suggests that O_2_ chemosensing in these cells is equivalent to other species and thus supports the idea of a highly conserved mechanism [17]. Whether CO_2_ chemosensing is equivalent cannot be determined, although we do show the presence of one key cytoplasmic CA, CAII. Since we have evidence that NEBs in other species are responsive to hypercapnia by releasing 5-HT, it will be interesting to determine if NMR NEB cells have similar sensing characteristics, or given their natural microenvironment, an alternate threshold for CO_2_ sensing. It is possible that NMR NEBs could be more insensitive to hypercapnia.

We also studied the expression of the hypoxia sensitive transcriptional machinery that involves the hypoxia inducible factors HIF1α and HIF2α [27], [28]. We reasoned that their relative hypoxic microenvironment could affect this system. Indeed we found that native lung tissue did not express any appreciable amounts of HIFs, however, when placed into culture (known to be an environment of relatively mild hypoxia) HIF1α and HIF2α were upregulated. Interestingly, only a fraction of the cells within a colony of 5-HT positive neuroendocrine cells were HIF positive. Moreover, finding HIF1α nuclear staining suggested functional activation unlike HIF2α that was essentially localized cytoplasmically. Previous studies have suggested that HIF1α and HIF2α differentially mediate acute and chronic hypoxia respectively [29]. One would need to determine if HIF2α would be upregulated under the chronic hypoxic environment experienced by NMR in their native habitat. Nevertheless, these observations suggest that the NMR NEB cells could undergo critical transcriptional programming events under hypoxia [27].

The neurogenic transcription factor ASH-1 (MASH1 in mouse, hASH1 in humans) is obligatory for PNEC/NEB development but then diminishes during postnatal and then into adult life [26]. We found that MASH1 was robustly expressed in NMR NEBs and localized to nuclei suggesting an active developmental state. The developmental state of the NMR NEB cells therefore suggested their resemblance to the fetal and neonatal functional states even in older animals. We note that the oldest NMRs in the present study were 3 months old, and we do not yet know if our findings will prove to be consistent over the extensive lifetime of this species. However, a similar conclusion that even older NMRs (>1 year) maintain a more fetal like developmental state has come from studies on the CNS [13] where naked mole-rat brain utilizes the neonatal NMDA receptor subunit, associated with hypoxia tolerance, and a blunted neuronal calcium response to hypoxia normally seen in neonatal mammals [14]. Great attention has been paid to the hypoxia tolerance of NMRs that also show an impressive resistance to oxidative stress which is attributed to other cytoprotective mechanisms since they possess a low level of antioxidant enzymes [29]. Whether this belies their impressive longevity can be only speculated upon at this time but does imply a potential greater degree of functional plasticity and therein adaptability.

The observation of increased size and number of NMR NEBs relative to WR also corresponds to the hyperplasia seen in humans and other animal species whether due to high altitude, lung disease with build up of hypoxia, or in a mouse model deficient in PHD-1, the O_2_ sensitive interacting protein that leads to degradation of HIF1α [30]. PNEC, normally exhibit a very low turnover [17]. PNEC are either triggered into cycle or there is recruitment and differentiation of progenitors [22], all perhaps leading to an increase in chemosensory capacity. In the case of the NMR NEBs studied here, they appear to reside in a more fetal state, but also robustly express the key components of the O_2_ sensor complex in NMR NEBs (gp91^phox^, p22^phox^ and Kv channels), all localized at the apical plasma membrane. We therefore surmise that NEB cells in the NMR are robustly functionally attuned to the hypoxic/hypercapnic state of their natural environments. In tune with this heightened sensory state we found NMR NEBs to be innervated by the multiple efferent and afferent fibers, cholinergic, purinergic and nitrergic, as seen in rat NEBs [24]. However, it should be noted that for the most part these contacts were few suggesting the presence of specialized sensor cells within the NMR NEBs. A few prominent P_2×2_ cells were noted in the NEBs supporting the idea of functional sub-specialization. Along this line the finding that only some cells within a NMR NEB turn on HIFs also supports this idea. Thus it would appear that NMR NEBs constitute a mix of specialized cells serving individual specific sensor functions. This then begs the question whether these different NEB cells within the complex subserve specific functions and, if they then communicate via Cx43 positive gap junctions to permit functional coordination.

A recent study suggests that PNEC cells are functionally receptive to odorants [31] by releasing 5-HT and CGRP. As these are also triggered by hypoxia/hypercapnia it would be interesting to determine if such odorant sensing systems are strongly expressed by specific cells within NMR NEBs since these animals live under extreme odorant conditions as well (e.g. ammonia). NEBs within the lung show evidence of being interconnected [17], [23], [24] via nerve networks into what could be considered to constitute a ‘brain’ like structure. One could therefore entertain the notion that the entire PNEC/NEB system in lung works cooperatively with the CNS to monitor the environments within the interstices of the lung and thereby alert the CNS to facilitate protection of the CNS against external toxicities. Given all the other unique properties of NMRs one could envision that this function is maximized in the NMR.

Although there is still debate on the physiological functional significance of NEBs, whether as O_2_ chemosensors or as pain sensors [18], other functions are now emerging [31]. All the building evidence supports the new idea that NEBs indeed could be multimodal sensors with exquisite sensitivity to the outside environment. Emerging evidence touting purines as clinically important mediators in the pro-inflammatory or protective responses [32] also lend further support to a possible key function in the NMR airways and our evidence vis a vis pannexin-1 staining suggests NMR NEBs may have some resistance this way. As presented here, it is possible, given the innervation complexity noted for NMR NEBs, that individual cells within the NEBs could also assume discrete sensory functions relayed through appropriate nerve fibers but integrated at the whole NEB level. Finally, as it is evident that amongst many species NMR live in an extreme microenvironment with multiple challenges to the airways we believe that the NMR model will help to decipher the, as yet, unrevealed complexity of the NEB sensory system. Such understanding could help humans to be able to adjust favorably to their rapidly changing environments.
